# Response-Category Conflict and Control Mode Determine Memory Performance for Distractors in a Flanker Paradigm

**DOI:** 10.5334/joc.338

**Published:** 2024-01-10

**Authors:** Michèle C. Muhmenthaler, Beat Meier

**Affiliations:** 1 Institute of Psychology, University of Bern, CH

**Keywords:** Flanker paradigm, response-category conflict, attentional boost hypothesis, attentional selectivity hypothesis, control mode

## Abstract

Cognitive conflicts can lead to better memory for task-relevant information. However, little is known about memory performance for task-irrelevant information. The present study investigated memory performance of task-irrelevant distractors using a Flanker paradigm. In two experiments (N = 699), participants first completed a study phase with 56 flanker trials. The stimuli consisted of trial-unique pictures. A congruent trial consisted of a target flanked by two identical pictures from the same category (e.g., all mammals). An incongruent trial consisted of a target and flankers from different stimulus categories (e.g., a mammal flanked by two identical birds), which results in a response-category conflict. To explore the impact of different control modes, congruent and incongruent trials were presented in pure blocks (allowing a proactive control mode) or in mixed blocks (requiring a reactive control mode). Afterwards, recognition memory was tested in a surprise memory test. In general, the results showed better memory for congruent than incongruent flankers in pure blocks. In contrast in mixed blocks, the results showed better memory for incongruent than for congruent flankers. Thus, memory performance for distractors varies systematically with response-category conflict and control mode.

To achieve our internal goals, it is essential to shield our performance from distracting information (Goschke & Dreisbach, 2008). Suppressing irrelevant distractors efficiently is costly and depends on the specific control demands of the task. According to Braver (2012), cognitive control operates through two distinct operating modes: “proactive control” and ”reactive control”. In the proactive control mode, the occurrence of distracting events can be anticipated by actively maintaining goal-relevant information. In the reactive control mode, attention is recruited only as needed, to respond after distracting events have occurred. Regardless of the control mode applied, distracting information has a negative impact on immediate task performance. Interestingly, the presence of distracting information can boost subsequent memory for *task-relevant* information (Davis et al., 2020; Jiménez et al., 2020; Krebs et al., 2015; Muhmenthaler & Meier, 2021a; Ptok et al., 2021; Rosner et al., 2015). However, little attention has been drawn to memory performance of the distracting, task-irrelevant information. The aim of the present study was to investigate memory performance for distractors systematically by using a flanker paradigm.

The flanker task has originally been used to investigate the impact of irrelevant information (i.e., flankers) on the processing of relevant information (i.e., targets). In the traditional flanker task, participants are asked to respond to a target stimulus surrounded by flanker stimuli on each side (Eriksen & Eriksen, 1974). Letters or arrows are often-used stimuli, and in the incongruent condition, the participants have to resolve the conflict between two potential responses (e.g., “ABA”). This leads to slower and less accurate responses than the congruent condition (e.g., “AAA”).

In a recent study, we used trial-unique pictures as targets and flankers in a study phase (Muhmenthaler & Meier, 2021b). Specifically, we used pictures of *one* category as target and flankers in the congruent condition (e.g., different birds), and we used pictures of *different* categories in the incongruent condition (e.g., a bird flanked by mammals). We labeled the emerging conflict between the response options for target and flanker “response-category conflict” (Muhmenthaler & Meier, 2021a). In a subsequent test phase, we assessed recognition memory performance for the pictures. The results showed better memory performance for incongruent compared to congruent targets, in line with similar conflict studies (Davis et al., 2020; Krebs et al., 2015; Muhmenthaler & Meier, 2021a; Ptok et al., 2019; Rosner et al., 2015, but see Jiménez et al., 2020). This encoding benefit under high-conflict conditions was interpreted in terms of the conflict-monitoring model, which postulates that cognitive control resolves conflict through selective attention mechanisms to bias perceptual processing toward task-relevant information (Botvinick et al., 2001; Egner & Hirsch, 2005). In other words, when a cognitive conflict occurs, attention is up-regulated and selectively directed to the targets.

However, it is not clear how this up-regulation of selective attention affects the processing of the task-irrelevant distractors. In most of the conflict studies memory for distractors was not reported (Davis et al., 2020; Jiménez et al., 2020; Krebs et al., 2015; Rosner et al., 2015). The reasons were methodological, as the distractors were repeatedly used words (Krebs et al., 2015; Ptok et al., 2019), presented simultaneously with the targets in the test, or were simply not included in the test (Davis et al., 2020; Rosner et al., 2015). It is thus unknown whether the task-irrelevant distractors are ignored, inhibited, or even strengthened when a conflict is detected. Notably, in our recent study, incongruent flankers were somehow better remembered than congruent ones, but the effect was not statistically significant (Muhmenthaler & Meier, 2021b). However, we acknowledge that the sample size was small, resulting in low statistical power for the subtle memory effects of the flankers. The current study was designed to investigate memory performance for flankers systematically. Toward this goal, we replicated and extended the experiment with a large sample.

We reasoned that the presence of response-category conflict might lead to *enhanced* flanker encoding, due to a transient up-regulation of attention when a conflict occurs (Botvinick et al., 2001; Egner, 2008; Egner & Hirsch, 2005). This up-regulation could lead to a broad focus of attention which strengthens encoding for all items in close spatiotemporal proximity to the target. In other terms, the incongruent flankers could “benefit” from the attentional boost associated with conflict (LaPointe et al., 2022; Swallow & Jiang, 2010; Verguts & Notebaert, 2009). Alternatively, it is also possible that the presence of response-category conflict leads to *reduced* flanker encoding, due to a narrowed focus of attention to shield from the distractors (Goschke & Dreisbach, 2008). Both outcomes are possible, dependent on the control mode which is elicited by the specific control demands of the task (Braver, 2012).

In order to manipulate the control mode, we presented the flanker stimuli in pure or in mixed blocks at study (Braver, 2012; Los, 1996; Rosner & Milliken, 2015). We reasoned that when response-category conflict is present in mixed blocks, a reactive control mode is necessary, and performance is stimulus driven (Braver, 2012). In this control mode, attention is recruited as a late correction mechanism that is mobilized in a just-in-time manner (Braver, 2012). In mixed blocks, the participants usually abstain from trial-specific preparations (Los, 1996, 1999). The emerging conflict between the response categories in incongruent trials may lead to an “attentional boost” which strengthens encoding for all items which are activated at the moment, even when they are task-irrelevant (Jiménez et al., 2022; LaPointe et al., 2022; Swallow & Jiang, 2013; Verguts & Notebaert, 2008, 2009). Accordingly, we hypothesized better memory for incongruent than congruent flankers in mixed blocks. We labeled this hypothesis the “attentional boost hypothesis”. This pattern of results would be consistent with our recent study in which we only included mixed blocks (Muhmenthaler & Meier, 2021b).

In contrast, when response-category conflict is manipulated in pure blocks (i.e., one congruent, one incongruent), a proactive control mode is invoked (Braver, 2012). The proactive control mode allows an early selection, leading to goal-driven processing, which affects selective attention. In incongruent blocks, goal-driven processing *enhances* selective attention to the targets and reduces attention to the flankers, which would activate an incorrect response (Goschke & Dreisbach, 2008). In congruent blocks however, goal-driven processing allows increased attention to the flankers, as they belong to the same stimulus category and may help to select the correct response (Muhmenthaler & Meier, 2021a). The congruent condition thus *reduces* selective attention. Accordingly, we hypothesized lower memory performance for incongruent than for congruent flankers. We labeled this hypothesis the “attentional selectivity hypothesis”.

## The present study

**Figure 1:**
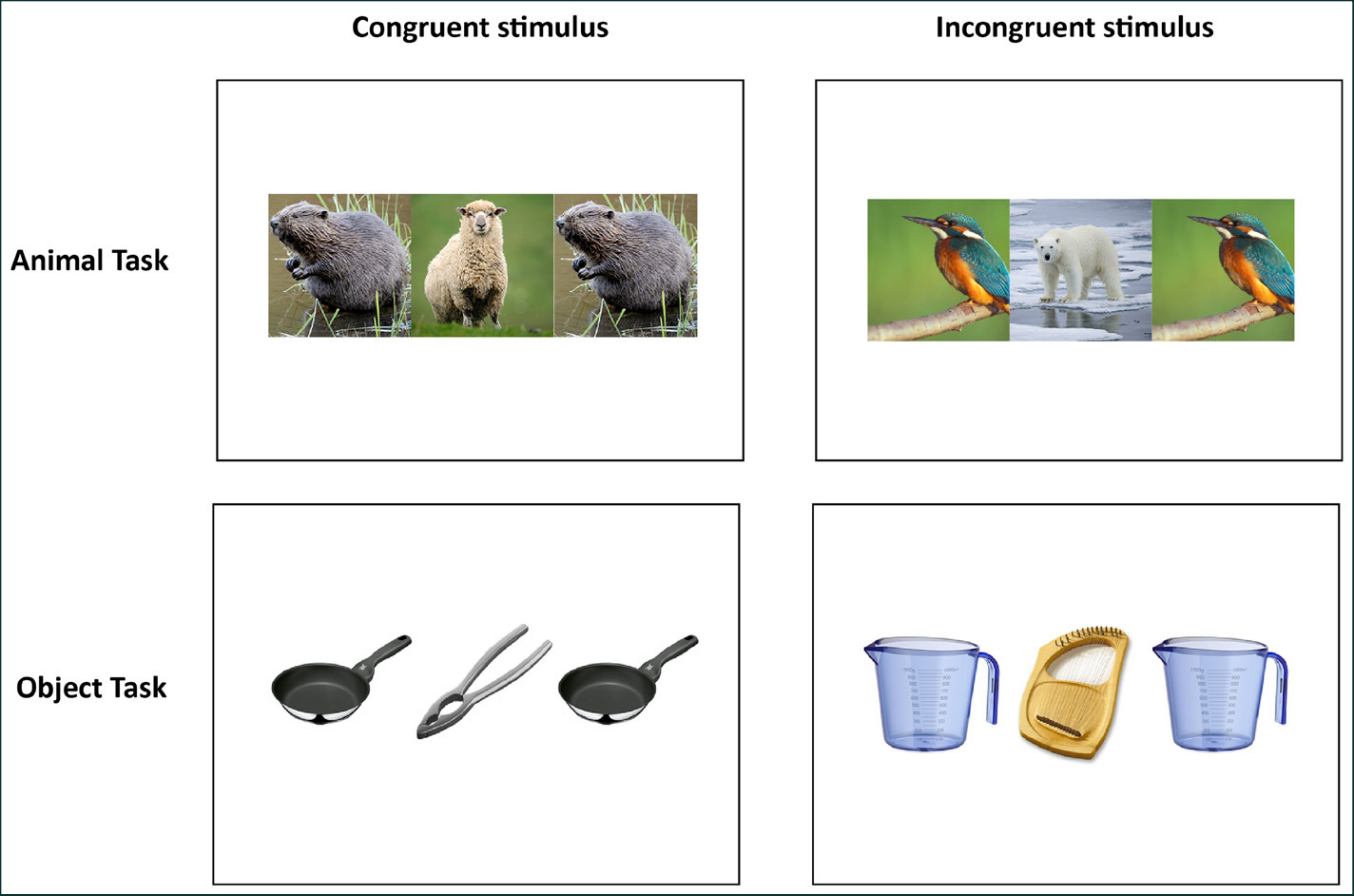
Left column: Congruent trial with a target and two flankers; all items derive from the same category. Right column: Incongruent trial, the target, and the flankers derive from different categories.

In order to test these hypotheses, we used trial-unique picture stimuli at study (cf. Muhmenthaler & Meier, 2021b). The participants had to classify pictures of animals (i.e., mammal vs. bird) and pictures of objects (i.e., musical instruments vs. kitchen utensils), see Figure 1. Congruent and incongruent trials were shown in mixed blocks for half of the participants. For the other half, congruent and incongruent trials were presented in pure blocks. A congruent trial consisted of *different* target and flanker objects from the *same* category (e.g., a dog, flanked by two monkeys). An incongruent trial also consisted of different targets and flanker objects but from *different* categories (e.g., a bear, flanked by two ducks). The reason was that in the incongruent condition of a traditional flanker task, two sources of interference are confounded: An incongruent trial (e.g., “BAB”) involves competition between conflicting response tendencies, as two potential responses for “A” and “B” are simultaneously present, and – compared to the congruent condition – involves higher perceptual load (Lavie et al., 2009; Sanders & Lamers, 2002; Yeh & Eriksen, 1984). With our setting, we made sure that potential memory effects rely on the cognitive conflict between target and flanker, and not on the differences in perceptual appearance.

## Experiment 1

### Method

#### Participants

In Experiment 1, 340 participants (*M* = 23.25 years, *SD* = 5.66, 150 males, 189 females, one other) were recruited and tested by undergraduate students. In an a priori power analysis (Cohen, 2013), we computed the sample size as a function of a power level of .95, a significance level of 0.05, and the expected effect size for the flankers. We took the non-significant effect size for the flankers from our prior work (*d* = 0.24) and calculated the minimal sample size using a two-tailed *t*-test. The resulting analysis computed a number of 228 participants. As part of a research method class, we were able to test 340 participants. The local ethical committee approved the study, and all participants gave written consent.

#### Materials

A stimulus consisted of two different pictures, a target in the middle, and two identical flankers. The size of each picture was 12 × 12 mm for half of the participants and 36 × 36 mm for the other half of the participants. We manipulated the pictures size, as the size was not standardized in our previous study. As the size of the stimuli did not affect immediate task or memory performance, we do not report this any further. In Experiment 1, there was no distance between target and flanker; the visual angle between target and flanker was therefore 0°.

For the experimental trials, the material consisted of 168 colored photographs of typical examples from the categories of mammals, birds, musical instruments, and kitchen utensils, 42 per category (cf., Muhmenthaler & Meier, 2021b). Two-thirds of the pictures (112 pictures, 28 per category) were used both for the study and the test phase. Fifty-six pictures were used as targets, and 56 were used as flankers. The remaining third (56 pictures, 14 per category) were used as lures. The trials were either congruent (a target and two flankers from the same category) or incongruent (a target and two flankers from different categories), an example is depicted in Figure 1. Half of the trials consisted of three pictures of animals, the other half of three pictures of objects. For the animal task, pictures of birds and pictures of mammals were used as flankers. For the object task, pictures of musical instruments and pictures of kitchen utensils were used as flankers. The animal pictures had colored backgrounds, whereas the objects were presented on a white background. Eight additional pictures were used for practice, two per category, which resulted in four practice trials.

#### Procedure

##### Study phase

**Figure 2:**
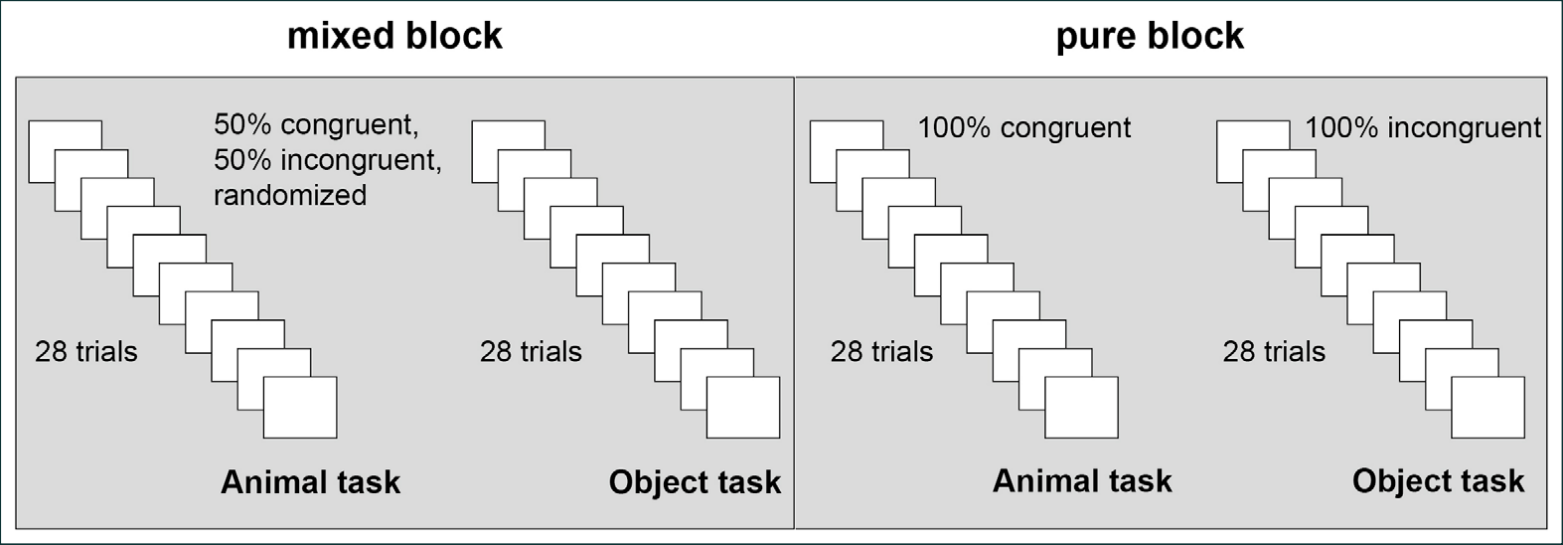
Experimental conditions. *Note*: Task order (both blocks) and congruence of the tasks (pure block) were counterbalanced across participants. Congruence was manipulated within participants, the factor block was manipulated between the participants.

Participants were tested individually. After giving consent, they were instructed to classify the picture in the center and ignore the two flanker pictures on the left and right as fast and accurately as possible. For the animal task, participants had to classify the target as mammal or bird; for the object task, they had to classify the target as a musical instrument or kitchen utensil. The two tasks were presented in two separate blocks. The participants were randomly assigned to the pure or mixed block condition. In the pure block condition, all the trials of a task block were either congruent or incongruent. Congruence of the task and task order were counterbalanced across participants. In the mixed block condition, task order was counterbalanced across participants. Within the blocks, congruent and incongruent trials were presented intermixed, half of the trials were congruent, the other half incongruent, see Figure 2.

After a practice phase with two trials, participants performed the animal or the object task with 28 trials; they were not instructed to memorize the pictures. Then, they were asked to perform the other task with two practice trials and 28 experimental trials. The participants responded on a standard computer keyboard using their index fingers. They had to press the *a*-key when the target picture was a mammal or a musical instrument and the *l*-key when the target picture was a bird or a kitchen utensil. The trials were presented until a response-key was pressed, then the next trial was presented after 200 ms of a blank screen. All the pictures appeared only once in the study phase. Following the study phase, participants counted backward aloud by seven from 300 for a maximal time of three minutes. The primary purpose of this task was to create a filled retention interval between the study and test phases.

##### Test phase

The third part of the experiment involved a surprise recognition memory test and an additional *remember/know* judgment (Tulving, 1985; Yonelinas, 2002). Participants had to indicate whether a picture was old or new (not seen in the study phase). All 56 targets, 56 flankers, and 56 lures were presented solely in the middle of the screen. They had to press the *j* –key for “old” pictures and the *n*-key for “new” pictures. In case of an “old”-response, they were required to give an additional *remember/know* judgment by pressing the *1*-key for “remember” or the *2*-key for “know” on the number pad. They were instructed to give a “remember” response when they were sure that they had seen the picture and to provide a “know” response when they perceived a feeling of familiarity. For each trial, the picture was presented until a response-key was pressed. The pictures appeared in randomized order with a response-stimulus interval of 200 ms. The entire experiment lasted about 25 minutes.

##### Design

The experiment included the factor response-category conflict (congruent vs. incongruent) as a within-subject factor, and the factor block (pure vs. mixed) as a between-subject factor, resulting in a 2 × 2 mixed design. The dependent variables were reaction times (RTs) and accuracy in the study phase, and hits (proportion of correctly remembered pictures) in the test phase.

### Results

#### Statistical analyses

We excluded participants with a study phase accuracy below .70 (cf., Muhmenthaler & Meier, 2019). Twenty-five participants were excluded due to this criterion, and 315 were included for the analyses. For the study phase, we conducted separate analyses on accuracy and reaction times (RTs) to test for a potential flanker effect. We excluded trials with reaction times below 150 ms and longer than 1500 ms (7% of the trials). To analyze accuracy and RTs, we then tested the flanker effect with a 2 × 2 mixed analysis of variances (ANOVA) using the trimmed data.

For the analyses of the test phase, in order to control for appropriate performance, we only included participants with a false alarm rate below 50% (cf., Muhmenthaler & Meier, 2021b). This resulted in a sample of 302 participants. We first conducted a one-way ANOVA to check if performance was above chance for the three picture types targets, flankers and false alarms, followed by post-hoc tests when appropriate. Next we analyzed response-category conflict (congruent vs. incongruent) and control mode (pure vs. mixed) using 2 × 2 ANOVAs. Due to highly significant sphericity tests, we conducted separate ANOVAs for the targets and the flankers. The false alarms were not included in these analyses as they cannot be assigned to the factor response-category conflict. All follow-up tests were one-sided due to the directed hypotheses. An alpha level of .05 was used, and effect sizes were reported as \[\eta _p^2\] or Cohen’s d.

#### Study phase

The 2 × 2 ANOVA with the factors response-category conflict (congruent vs. incongruent) and block (pure vs. mixed) showed slower RTs for incongruent (*M* = 725 ms, *SE* = 6 ms) than congruent trials (*M* = 715 ms, *SE* = 6 ms), *F*(1, 313) = 4.13, *p* = .043, \[\eta _p^2\] = .01. The factor block was not significant, *F*(1, 313) < 1, *p* = .483, \[\eta _p^2\] < .01. The interaction between response-category conflict and block reached significance, *F*(1, 313) = 6.83, *p* = .009, \[\eta _p^2\] = .02. In the mixed block, the RTs of the congruent trials (*M* = 717 ms, *SE* = 8 ms) and the incongruent trials (*M* = 714 ms, *SE* = 12 8) did not differ, *t*(180) < 1, *p* = .504, *d* = 0.050. However, in the pure block, the RTs of congruent trials (*M* = 712 ms, *SE* = 9 ms) were faster than RTs of the incongruent trials (*M* = 735 ms, *SE* = 8 ms), *t*(133) = 2.32, *p* = .022, *d* = 0.200.

The same ANOVA on accuracy using trimmed data revealed that congruent trials (*M* = .96, *SE* = .004) were more accurate than incongruent trials (*M* = .94, *SE* = .003), *F*(1, 313) = 11.03, *p* = .001, \[\eta _p^2\] = .03. Pure blocks (*M* = .97, *SE* = .004) led to higher accuracy than the mixed blocks (*M* = .95, *SE* = .004), but the difference was not significant, *F*(1, 313) = 2.82, *p* = .094, \[\eta _p^2\] = .01. The interaction between response-category conflict and block was not significant, *F*(1, 313) = 2.16, *p* = .142, \[\eta _p^2\] = .01.

#### Test phase

##### Targets, flankers and false alarms

The one-way ANOVA on picture type with the three levels target, flanker and false alarms was highly significant, *F*(1, 602) = 2054, *p* < .001, \[\eta _p^2\] = .87. Separate *t*-tests revealed that the mean hit rate for targets (*M* = .676, *SE* = .011) exceeded the mean hit rate for flankers (*M* = .240, *SE* = .008), *t*(301) = 42.12, *p* < .001, *d* = 2.41. The mean hit rate for the targets also exceeded the mean false alarm rate (*M* = .205, *SE* = .008), *t*(301) = 50.31, *p* < .001, *d* = 2.72. Importantly, the mean hit rate for flankers exceeded the false alarm rate, *t*(301) = 8.82, *p* < .001, *d* = 0.475, indicating that flanker memory was above chance level.

##### Targets

Descriptives of the targets across conditions are presented in Table 1. For The 2 × 2 ANOVA on response-category conflict (congruent vs. incongruent) × block (pure vs. mixed) revealed that more incongruent targets were remembered (*M* =.683, *SE* =.012) than congruent targets (*M* =.655, *SE* =.012), *F*(1, 300) = 10.75, *p* = .001, \[\eta _p^2\] = .03. The factor block was not significant, *F*(1, 300) < 1, *p* = .390, \[\eta _p^2\] < .01. The interaction between response-category conflict and block was significant, *F*(1, 300) = 5.19, *p* = .023, \[\eta _p^2\] = .02. In the pure block, more incongruent targets (*M* = .682, *SE* = .018) were remembered than congruent targets (*M* = .636, *SE* = .018), *t*(133) = 3.14, *p* =.033, *d* = 0.271. In the mixed block, the difference between incongruent (*M* = .683, *SE* = .016) and congruent targets (*M* = .675, *SE* = .016) was not significant, *t*(170) = 1.19, *p* = .118, *d* = 0.236.

**Table 1:** Means and standard deviations (in brackets) for hit rates of flankers and targets, separately for each condition, and for both experiments.


		MEANS (STANDARD DEVIATION)			

		PURE		MIXED	
	
		CONGRUENT	INCONGRUENT	CONGRUENT	INCONGRUENT

Experiment 1	Flanker	.263 (.172)	.235 (.138)	.224 (.124)	.258 (.139)

	Target	.636 (.213)	.682 (.192)	.675 (.214)	.683 (.215)

Experiment 2	Flanker	.252 (.145)	.236 (.139)	.200 (.121)	.245 (.140)

	Target	.697 (.177)	.730 (.174)	.662 (.198)	.665 (.188)


##### Flankers

**Figure 3:**
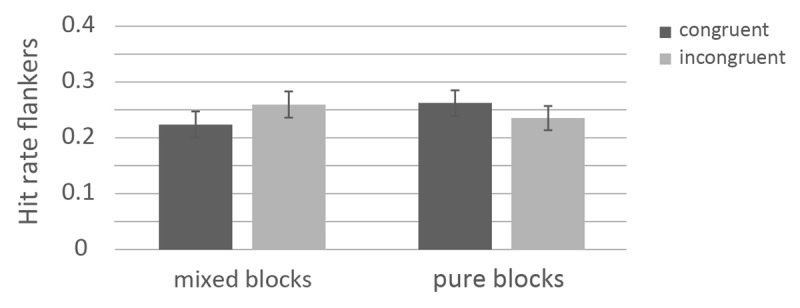
Memory performance for flankers as a function of response-category conflict and control mode. *Note*: The error bars represent standard errors.

Descriptives of the flankers across conditions are presented in Table 1. The 2×2 ANOVA on response-category conflict (congruent vs. incongruent) × block (pure vs. mixed) revealed that overall, congruent (*M* = .243, *SE* = .009) and incongruent flankers (*M* = .247, *SE* = .008) did not differ, *F*(1, 313) < 1, *p* = .489, \[\eta _p^2\] < .01. The factor block was not significant (mixed: .241, *SE* = .011, pure: .249, *SE* = .011), *F*(1, 313) < 1, *p* = .859, \[\eta _p^2\] < .01. The interaction between response-category conflict and block was significant, *F*(1, 313) = 15.56, *p* < .001, \[\eta _p^2\] = .05. In the blocked context, more congruent flankers (*M* = .263, *SE* = .015) were remembered than incongruent flankers (*M* = .235, *SE* = .012), *t*(131) = 1.78, *p* =.030, *d* = 0.165. In the mixed context, more incongruent flankers (*M* = .258, *SE* = .010) were remembered than congruent ones (*M* = .224, *SE* = .010), *t*(170) = 4.04 *p* < .001, *d* = 0.309. The results of the ANOVA on the flankers is depicted in Figure 3.

### Discussion

The results of the study phase showed flanker effects in terms of RTs and accuracy. Incongruent trials led to slower RTs than congruent trials. RTs for mixed and pure blocks did not differ, but in the mixed blocks, participants were somewhat less accurate. In mixed blocks, performance is usually slower than in pure blocks, even when the blocks are purely incongruent (Los, 1996). The lack of mixing costs in terms of RTs might reflect a speed-accuracy trade-off: Performance in purely incongruent blocks was slower than in mixed blocks, but also more accurate. The results imply that the participants applied context-sensitive control modes (Braver, 2012; Rosner & Milliken, 2015).

The data of the memory test revealed that memory performance for the flankers varied systematically with response-category conflict and control mode. When response-category conflict was manipulated in pure blocks, triggering a proactive mode of control, the results showed better memory for congruent than incongruent flankers. In the incongruent block, attention was focused toward the targets, thereby inhibiting the flankers. In contrast in the congruent condition, there was increased attention to the flankers, as they triggered the same response (the flanker could be a more typical exemplar of the category). However, this attentional spread from target to flankers not only improved flanker memory, but also reduced target memory. This is evidence for the “attentional selectivity hypothesis”: Selective attention was increased in the presence of a response-category conflict, but decreased when this conflict was absent.

When response-category conflict was present in mixed blocks, triggering a reactive mode of control, the opposite was true: The results showed better memory for incongruent than congruent flankers. This finding implies that attention was boosted in incongruent trials, leading to enhanced encoding for the items involved in the conflict trial (LaPointe et al., 2022; Mulligan et al., 2014; Swallow & Jiang, 2013; Verguts & Notebaert, 2008, 2009). However, memory for incongruent targets was only numerically enhanced. This outcome was unexpected, as in our recent study, we found better memory performance for incongruent targets by using a very similar procedure. Notably the only difference was that the size of the individual pictures was not controlled as rigorously as in the present study. Paradoxically, in our previous study, the effect size for the flankers was smaller and not significant.

We reasoned that the larger memory effect of the flankers in mixed blocks - and the lack of a memory effect of the targets – could be attributed to the appearance of the stimuli: The target and the flankers were presented next to each other without any separation. It is conceivable that the participants perceived the stimuli as an entity rather than as a target flanked by two items. Consequently, a “spillover” from the targets to the flankers may have occurred (Ahissar & Hochstein, 2000). Miller (1991) found that irrelevant flanker letters still produced compatibility effects even when separated from relevant target letters by almost 5°. In a follow-up experiment, we enlarged the distance between the target and the flankers to test this possibility.

## Experiment 2

Experiment 2 was identical to Experiment 1, except that the target and flanker had a distance between them. The visual angle was around 3 degrees (depending on the size of the pictures).

### Method

#### Participants

In Experiment 2, 359 participants (*M* = 23.0 years, *SD* = 4.35, 192 males, 161 females, and two other) were recruited and tested by undergraduate students in the context of a research methods course. The local ethical committee approved the study, and all participants gave written consent.

#### Materials

**Figure 4:**
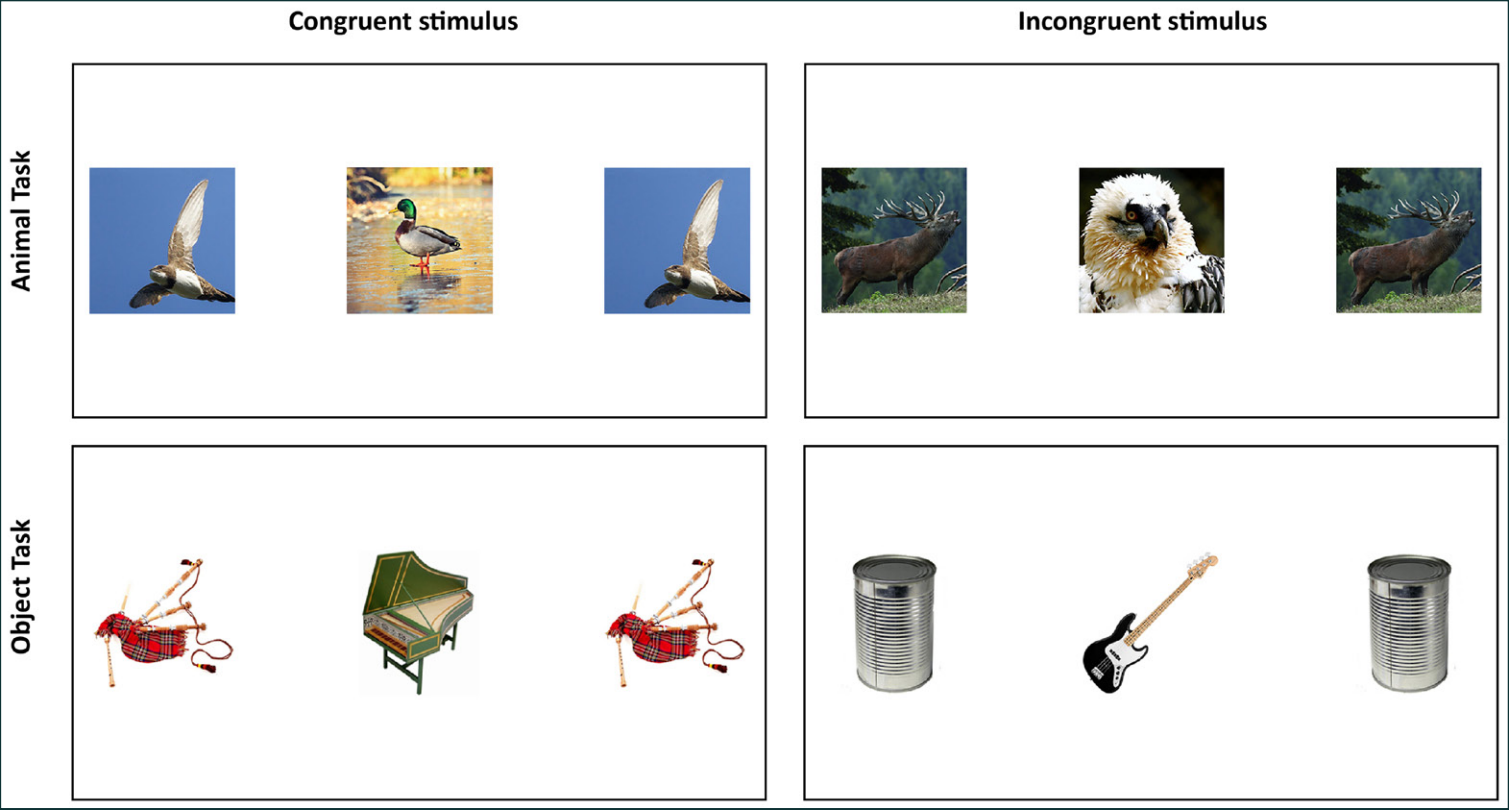
Left column: Congruent trial with a target and two flankers; all items derive from the same category. Right column: Incongruent trial, the target, and the flankers derive from different categories.

The materials were identical to Experiment 1 with the following exceptions. The large stimuli consisted of pictures of 36x36 mm. The distance between the target and flanker was 28 mm. The entire stimulus was therefore 36 × 111 mm, see Figure 4. The visual angle was 2.4°. Each picture of the small stimuli was 12 × 12 mm on the used laptops. The distance between the target and flanker was 43 mm. The whole stimulus was therefore 12 mm × 65 mm. The visual angle was 3.9°. As the size of the stimuli showed no effects, we do not report this factor any further.

#### Procedure

The procedure was identical to Experiment 1.

### Results

#### Statistical analyses

The analyses were identical to Experiment 1. For the analyses of the study phase, we excluded trials with reaction times below 150 ms and longer than 1500 ms (7% of the trials). We included participants with an accuracy of more than 0.70 in the study phase. This led to the exclusion of 28 participants, and the data of 331 participants were used for the analyses. For the test phase, we only included participants with a false alarm rate lower than 50%, which resulted in a final sample of 308 participants.

#### Study phase

For the analysis of RTs, the 2 × 2 ANOVA on response-category conflict (congruent vs. incongruent) and block (mixed vs. blocked) showed slower RTs for incongruent (*M* = 766 ms, *SE* = 11 ms) than congruent trials (*M* = 749 ms, *SE* = 9 ms), *F*(1, 329) = 7.55, *p* = .006, \[\eta _p^2\] = .01. The results showed slower RTs for the mixed (*M* = 794, *SE* = 14) than the blocked context (*M* = 722, *SE* = 14), *F*(1, 329) = 14.2, *p* < .001, \[\eta _p^2\] = .04. The interaction between response-category conflict and block was not significant, *F*(1, 329) < 1, *p* = .801, \[\eta _p^2\] < .01. The same ANOVA on accuracy using trimmed data revealed that congruent trials (*M* = .96, *SE* = .013) and incongruent trials (*M* = .96, *SE* = .015) did not differ, *F*(1, 329) = 2.06, *p* = .152, \[\eta _p^2\] = .01. The factor control mode (*F*(1, 329) = 1.56, *p* = 212, \[\eta _p^2\] = .01) and the interaction between block and response-category conflict were not significant, *F*(1, 329) = 3.57, *p* = .060, \[\eta _p^2\] = .01.

#### Test phase

##### Targets, flankers and false alarms

The one-way ANOVA on the picture types with the tree levels target, flanker and false alarms was highly significant, *F*(2, 614) = 2255, *p* < .001, \[\eta _p^2\] = .88. The mean hit rate for targets (*M* = .691, *SE* = .010) exceeded the mean hit rate for flankers (*M* = .235, *SE* = .007), *t*(307) = 48.45, *p* < .001, *d* = 2.31. The mean hit rate for targets exceeded the mean false alarm rate (*M* = .215, *SE* = .006), *t*(307) = 52.28, *p* < .001, *d* = 2.51. Most importantly, the mean hit rate for the flankers (*M* = .235, *SE* = .007) exceeded the false alarm rate (*M* = .215, *SE* = .006), *t*(307) = 4.12, *p* < .001, *d* = 0.220, indicating that flanker memory was above chance level.

##### Targets

Descriptives of the targets across conditions are presented in Table 1. The 2×2 ANOVA with the factors response-category conflict (congruent vs. incongruent) and block (blocked vs. mixed) revealed that more incongruent targets were remembered (*M* =.697, *SE* =.010) than congruent targets (*M* =.679, *SE* =.011), *F*(1, 306) = 4.68, *p* = .031, \[\eta _p^2\] = .02. The pure block (*M* =.713, *SE* =.013) led to better memory performance than the mixed block (*M* =.663, *SE* =.014), *F*(1, 306) = 6.79, *p* = .010, \[\eta _p^2\] = .02. The interaction between response-category conflict and block was not significant, *F*(1, 306) = 3.22, *p* = .074, \[\eta _p^2\] = .01.

##### Flankers

**Figure 5:**
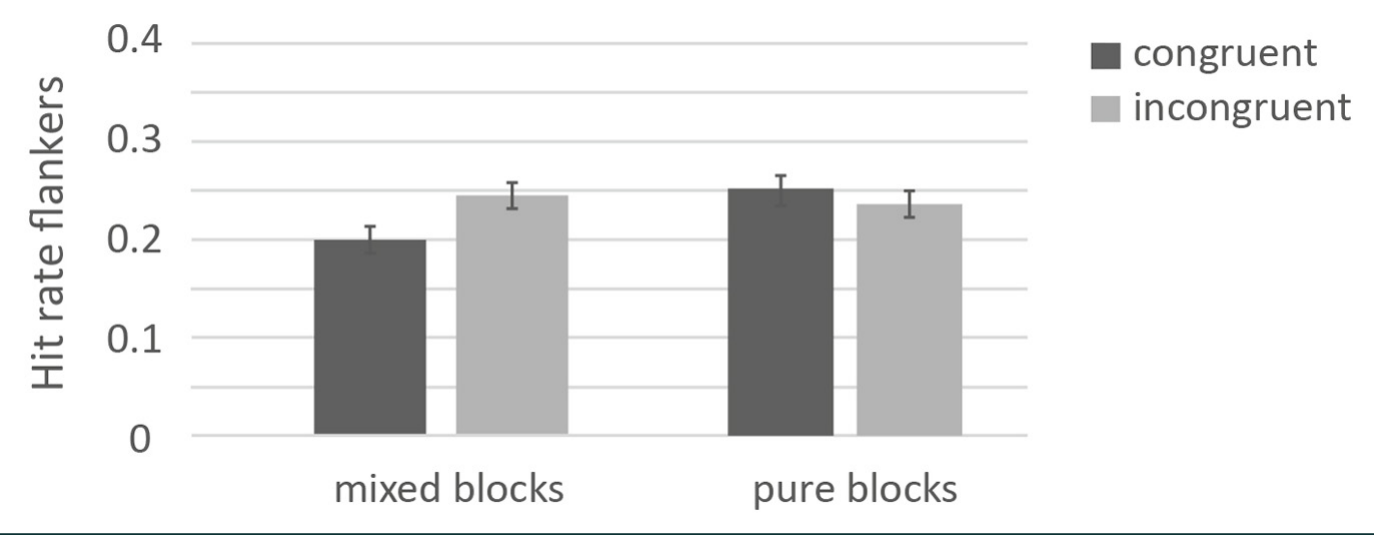
Memory Performance for the flankers as a function of response-category conflict and control mode in Experiment 2. *Note*: The error bars represent the standard errors.

Descriptives of the flankers across conditions are presented in Table 1. The 2×2 ANOVA on response-category conflict (congruent vs. incongruent) × block (blocked vs. mixed) revealed that memory performance for congruent (*M* = .226, *SE* = .009) and incongruent flankers (*M* = .240, *SE* = .009) did not differ significantly, *F*(1, 306) = 3.53, *p* = .061, \[\eta _p^2\] = .01. The factor block was not significant (mixed: .223, *SE* = 012; pure: .244, *SE* = .011), *F*(1, 306) = 2.39, *p* = .123, \[\eta _p^2\] = .01. The interaction between response-category conflict and block was significant, *F*(1, 306) = 16.32, *p* < .001, \[\eta _p^2\] = .05, the pattern of results is depicted in Figure 5. When response-category conflict was manipulated in pure blocks, the difference between the incongruent (*M* = .236, *SE* =.011) and the congruent block (*M* = .252, *SE* =.011) differed only numerically, *t*(172) = 1.48, *p* = .068, *d* = .110. When the blocks were mixed, incongruent flankers (*M* = .245, *SE* = .014) were better remembered than congruent flankers (*M* = .200, *SE*= .015), *t*(134) = 4.55, *p* <. 001, *d* = 0.392.

### Discussion

The study phase showed flanker effects and mixing costs regarding reaction times; the accuracy did not vary with conditions. The results imply that the participants applied context-specific control modes (Braver, 2012; Los, 1996, 1999; Rosner & Milliken, 2015).

When response-category conflict was present in pure blocks, the results showed better memory for congruent than incongruent flankers, but this difference was not statistically significant. In this setting, there was a large gap between target and flanker, making it less likely that the focus of attention spread to the flankers in the congruent block. This resulted in comparable memory performance for congruent and incongruent flankers. This hypothesis is supported by the observation that response times in the study phase were much longer in the purely incongruent block of Experiment 1 compared to Experiment 2.

In the mixed block, more incongruent than congruent flankers were remembered, providing evidence for the “attentional boost hypothesis” (LaPointe et al., 2022; Swallow & Jiang, 2013; Verguts & Notebaert, 2009). The attentional boost strengthened encoding of the whole conflict trial. In contrast, memory performance for congruent flankers was at chance level, providing evidence that they were not attended in the reactive mode.^1^ As in Experiment 1, the effect size for the flankers was larger than in our initial study, and we achieved high statistical power (>.99). Thus, the memory-enhancing effect for incongruent flankers seems quite robust, as it occurs despite a large gap between target and flanker.

## General Discussion

In order to investigate subsequent memory performance for distractors, we used a flanker task at study with trial-unique pictures in two experiments. Congruent and incongruent flanker stimuli were either shown in pure or mixed blocks, to manipulate the control mode (Davis et al., 2020; Jiménez et al., 2022; Rosner & Milliken, 2015; Muhmenthaler & Meier, 2021a; Whitehead et al., 2018). Proactive control relies on the anticipation of conflict before it occurs, whereas reactive control relies on the detection and resolution of conflict after it occurred (Braver, 2012). The results showed that memory performance for the task-irrelevant flankers varied systematically with the presence of response-category conflict and control mode.

Specifically when the flanker trials were presented in pure blocks, a proactive control mode was triggered, as the next trial can be anticipated (Braver, 2012; Los, 1996). The results revealed lower memory performance for incongruent than congruent flankers, accompanied by higher memory performance for incongruent than congruent targets. Thus, in the congruent block, memory for task-irrelevant information (i.e., flankers) was increased, whereas memory for task-relevant information was decreased (i.e., targets). This pattern resembles results from task-switching studies. Several authors showed that task switching affects subsequent memory performance (Chiu & Egner, 2016; Dubravac & Meier, 2022; Muhmenthaler et al., 2023; Richter & Yeung, 2012). In switch trials, selective attention is impaired due to enhanced control demands (Muhmenthaler & Meier, 2019; Rogers & Monsell, 1995; Wylie & Allport, 2000). As a consequence, memory for task-relevant information is reduced whereas memory for task-irrelevant distractors is enhanced, given that compound stimuli are used. Thus, more selective attention at study results in a “memory selectivity effect” (Dubravac & Meier, 2022; Lavie, 2010; Richter & Yeung, 2012). In the pure blocks of our study, a similar memory selectivity effect occurred.

When response-category conflict was present in mixed blocks, incongruent flankers were consistently better remembered than congruent flankers, providing evidence for the recruitment of attentional resources when a response-category conflict occurred. In congruent blocks, the flankers were processed rather automatically (no conflict detected), and thus less likely to be remembered than the incongruent flankers. An open question is whether enhanced flanker memory can be seen as a memory gain or a failure, as task-irrelevant materials intruded as a “by-catch” (Lavie, 2005).

Notably, both experiments showed a similar pattern regarding memory performance. However, regarding performance in the study phase, the pattern was not entirely consistent. The distance between target and flanker affected study task performance much more than subsequent memory. In line, in one of our recent studies, we found no congruence effects at study, but we found memory effects associated with congruence (Muhmenthaler & Meier, 2021a). Similarly, Rosner and Milliken (2015) reported two experiments in which conflict stimuli were presented in pure or mixed blocks. Immediate task performance resulted in comparable mixing costs in both experiments. However, when the stimuli were processed in pure blocks, memory effects occurred, which was not the case in mixed blocks. Accordingly, these results indicate that the memory test does not necessarily mirror exactly the outcome of cognitive control demands at study (Richter & Yeung, 2012).

It is also important to note that the effects sizes are generally small, as can be expected by the instructions to ignore the flanker stimuli. To tease out these subtle effect, we designed the present study with a large sample size. Overall, memory performance for the flankers exceeded the false alarm rate, implying that memory performance was nevertheless above chance level. Moreover, the consistent results across the two experiments indicate that although the effect sizes are small, they are reliable across experiments and studies (cf., Muhmenthaler & Meier, 2021b).^2^

The primary goal of this study was to disentangle differential effects of response-category conflict and control mode. However, it can be considered as a limitation that we did not include a neutral “congruence” baseline. As a consequence, it is not possible to decide whether in the pure blocks *increased* attention to the congruent flankers, *reduced* attention to the incongruent flankers, or both, caused the memory effect of the flankers. Thus, a follow up study should include neutral trials, that is, a ”congruence” baseline to decide between these possibilities.

## Conclusion

The present study investigated memory performance of task-irrelevant flanker pictures. Our experiments showed that the control mode elicited by the context at study determines whether the conflicting distractors are better or less well remembered than the non-conflicting ones. Proactive control (i.e., pure blocks) led to better memory for non-conflicting, congruent flankers, probably due to a shift of attention from the targets to flankers. Reactive control (i.e., mixed blocks) led to an attentional boost which enhanced memory for incongruent flankers, as a result of the emerging response-category conflict. Together, our results provide evidence that top-down and bottom-up mechanisms, elicited by the control mode and conflict at study, differentially contribute to the specific memory effects.

## Data Accessibility Statement

Data are available on the Open Science Framework at link: https://osf.io/jv735/.

## Footnotes

In the mixed block, a Bayesian *t*-test test provided evidence for a null effect between the hit rate for congruent flankers (*M* =.243, *SE* =.011) and the false alarm rate (*M* =.233, *SE* =.011), *BF* = 0.254, suggesting that these flankers were remembered at chance level.

Following a suggestion of a reviewer, we computed an ANOVA to compare memory performance of the flankers across the two experiments. The results showed that neither the additional factor «experiment» nor any interaction including this factor was significant.

## Open Practices Statement

The experiment reported in this article was not formally preregistered.
